# Conditional spatial classification of expert-confirmed interictal epileptiform discharge epochs: An EEG-ECG ablation and SHAP analysis

**DOI:** 10.1371/journal.pone.0358782

**Published:** 2026-09-22

**Authors:** Al Mukshit Plabon, Abdul Mukit, Md. Neyamul, Omar Faruk Jehady, Fatima Tuz Zuba, Md. Faisal Mina, Torikul Islam

**Affiliations:** 1 Department of Biomedical Engineering, Jashore University of Science and Technology, Jashore, Bangladesh; 2 Department of Biomedical Engineering, Khulna University of Engineering and Technology, Khulna, Bangladesh; 3 Department of Biomedical Engineering, New Jersey Institute of Technology, Newark, New Jersey, United States of America; Athinoula A Martinos Center for Biomedical Imaging, UNITED STATES OF AMERICA

## Abstract

Interictal epileptiform discharges (IEDs) are diagnostically important EEG abnormalities, but after an epoch has already been confirmed as containing IED, a separate methodological question remains whether its scalp distribution can be assigned among predefined categories, and do routinely co-recorded auxiliary channels alter that classification. This conditional five-class tasks were evaluated using 2,514 expert-confirmed four-second IED epochs labelled as generalized, frontal, temporal, occipital, or centro-parietal. Identical stratified epoch-level partitions, training-only SMOTE, 26 handcrafted features per included channel, and multiple machine-learning classifiers were used across a staged channel ablation comparing 19-channel scalp EEG, 21-channel EEG with two ECG channels, and the complete 29-channel input containing scalp EEG, referential, ECG, and EMG channels. Linear discriminant analysis achieved the best EEG-only test accuracy (88.89%), whereas CatBoost achieved 93.25% on EEG with ECG signals and 94.44% with the whole channel. All eight directly comparable classifiers showed numerically higher test accuracy after ECG was added, although these differences were descriptive and were not subjected to formal paired significance testing. SHAP analysis identified the key channel-feature variables influencing the fitted models, complementing performance ablation. In the EEG with ECG on CatBoost model, ECG channels RA and LA contributed 15.79% and 15.12% of normalized global attribution, respectively, while beta-band power was the most important feature family, accounting for 18.76%. The study therefore provides a controlled analysis of downstream spatial categorization and auxiliary-channel dependence within expert-confirmed IED epochs. The results do not establish physiological localization, unseen-patient generalization, or a clinically validated ECG biomarker.

## Introduction

Epilepsy is a neurological disorder characterized by recurrent unprovoked seizures arising from abnormal neuronal activity [1]. Electroencephalography (EEG) is central to clinical assessment because it records cerebral electrical activity and can reveal interictal epileptiform discharges (IEDs) between seizures [2,3]. Beyond the presence of an IED, its morphology and scalp distribution contribute to electroclinical description and annotation [4]. The scalp-distribution label, however, should not be equated with source localization, the seizure-onset zone, or the epileptogenic zone.

In the source dataset, expert-confirmed IEDs were organized into five predefined scalp-distribution categories: generalized, frontal, temporal, occipital, and centro-parietal [5]. Once an epoch has already been identified as containing an IED, assigning such a distribution label becomes a distinct downstream classification problem. Studying this conditional task is methodologically useful because it isolates discrimination among expert-defined spatial categories from the separate problem of deciding whether an IED is present at all. It can therefore provide a controlled benchmark for downstream annotation and for examining what recorded channel groups contribute to the separation of these labels. Throughout this article, “spatial classification” refers only to these predefined scalp-distribution categories and not to source reconstruction or localization of the seizure-onset zone.

IED detection and conditional spatial classification address different questions [6,7]. Detection evaluates whether unselected EEG contains an IED in the presence of non-IED background, artifacts, and IED-like transients [8]. The present analysis begins only after expert confirmation of IED presence and asks how the confirmed epoch is categorized among five scalp-distribution labels. This restricted design intentionally holds the detection decision constant so that channel-set and model effects can be examined within the IED-positive subset.

Machine learning provides a practical analytical framework for this task because each epoch is represented by hundreds of channel-wise time-domain, frequency-domain, and nonlinear descriptors, and the mapping from these variables to five labels may be multivariate and model-dependent. Evaluating several classifier families under identical data partitions allows the study to test whether the conditional categories are separable from the handcrafted representation and whether observed channel-set effects are consistent across different modelling assumptions. The purpose is not to replace expert IED detection, but to evaluate a high-dimensional downstream categorization problem after expert annotation.

The source recordings include scalp EEG together with referential, electrocardiogram (ECG), and electromyogram (EMG) channels [5]. ECG was evaluated because it is a simultaneously recorded auxiliary modality that can be added without changing the IED labels or scalp EEG channels. The EEG-versus-EEG with ECG comparison therefore asks a focused ablation question whether does the fitted classifier obtain additional discriminatory information when the two ECG channels are available. This test does not assume that any gain is autonomic or physiologically localizing; rather, it helps determine whether the model depends on information outside scalp EEG and highlights the need to consider about patient, state, or recording-specific structure when interpreting multimodal models. The subsequent full-input comparison jointly adds four referential and four EMG channels and consequently cannot separate their individual effects.

SHapley Additive exPlanations (SHAP) were incorporated to answer a complementary question to the channel ablation [9]. Ablation quantifies whether predictive performance changes when a channel group is added, whereas SHAP identifies which channel-feature variables a fitted model uses to produce its outputs. This combination makes SHAP central to the interpretability aim of the study: it can show whether an apparent performance gain is accompanied by substantial model dependence on the added modality and which features account for that dependence. Because SHAP importance is conditional on the dataset, feature representation, model, and target [10], the resulting attributions are interpreted only as model-specific predictive contributions, not as physiological biomarkers, causal mechanisms, or evidence of clinical localization [11]. This caution is especially important for ECG and EMG variables, which may reflect patient identity, sleep-wake state, recording conditions, movement, contamination, or other dataset-specific structure [12–14].

Accordingly, this study aimed to: (1) evaluate whether expert-confirmed IED epochs can be separated among five predefined scalp-distribution categories using a common handcrafted-feature representation; (2) compare multiple machine-learning classifiers under identical epoch-level partitions; (3) quantify the descriptive performance change when two ECG channels are added to 19-channel scalp EEG; (4) compare EEG with ECG using the complete 29-channel input; and (5) utilize SHAP to characterize model dependence on specific channels and features, while keeping physiological and clinical claims explicitly outside the scope of the analysis.

## Methodology

The study used a conditional spatial-classification pipeline restricted to expert-confirmed IED epochs in five predefined classes: generalized, frontal, temporal, occipital, and centro-parietal. The same preprocessing, channel-wise feature extraction, data partitions, imbalance handling, classifier evaluation, and SHAP framework were applied across channel configurations so that the ablation compared channel availability rather than different modelling pipelines.

### Proposed methodology

**Fig 1:**
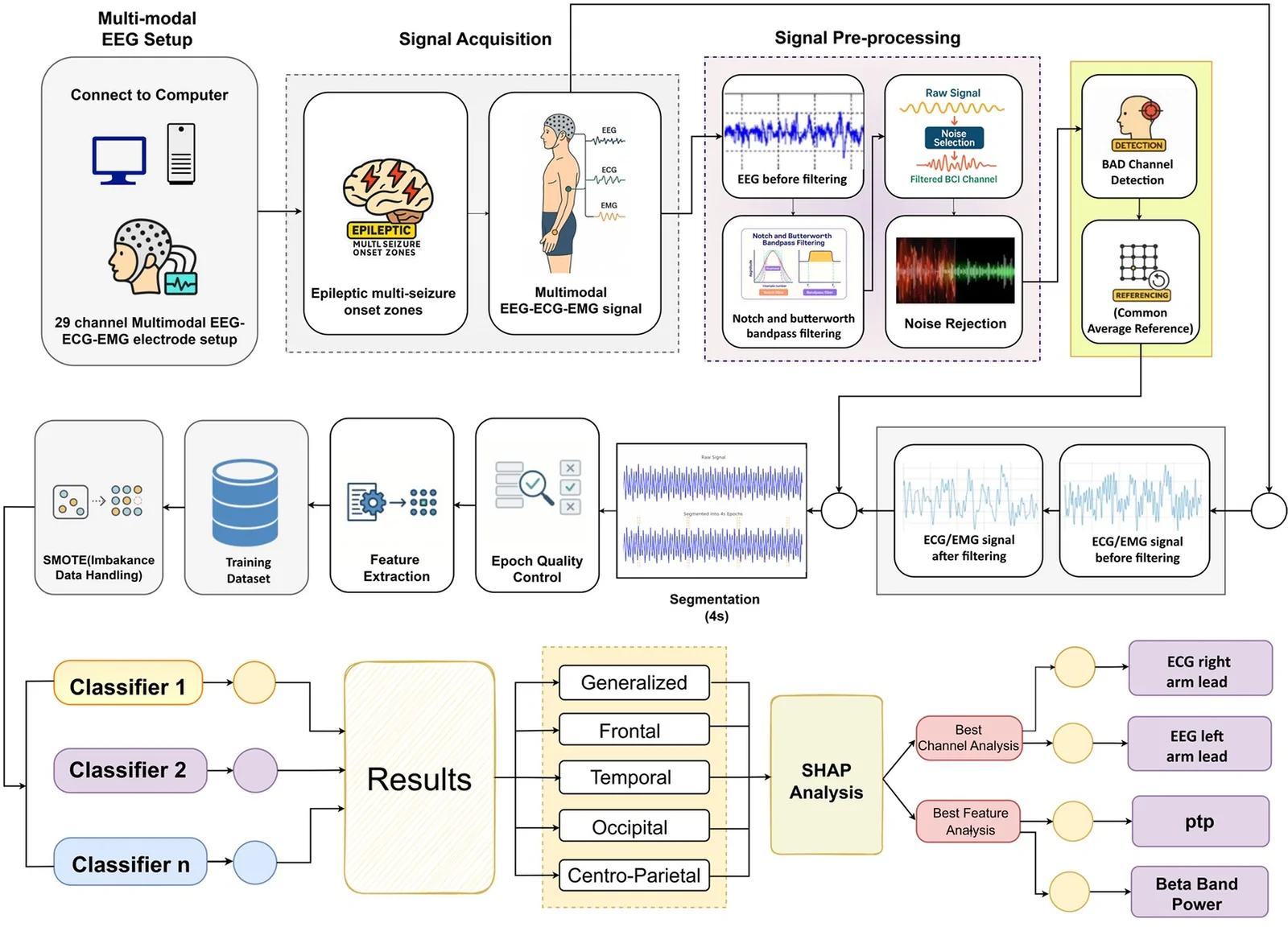
Study workflow for preprocessing, channel specific feature extraction, conditional five-class spatial classification, channel set ablation and SHAP analysis.

For the proposed work, the same expert-confirmed IED epochs followed the workflow shown in Fig 1. Twenty-six handcrafted features were extracted independently from every included channel. Three primary feature matrices were created: (1) 19 scalp EEG channels (19 x 26 = 494 variables); (2) 19 scalp EEG channels plus the two ECG channels, RA and LA (21 x 26 = 546 variables); and (3) the full 29-channel input comprising 19 scalp EEG, four referential channels, two ECG channels, and four EMG channels (29 x 26 = 754 variables). Each classifier received a flat vector of channel-feature variables. Electrode coordinates, adjacency matrices, inter-electrode distances, topographic maps, and source-reconstruction outputs were not used; spatial identity was represented only through channel-specific feature names.

### Dataset

**Fig 2:**
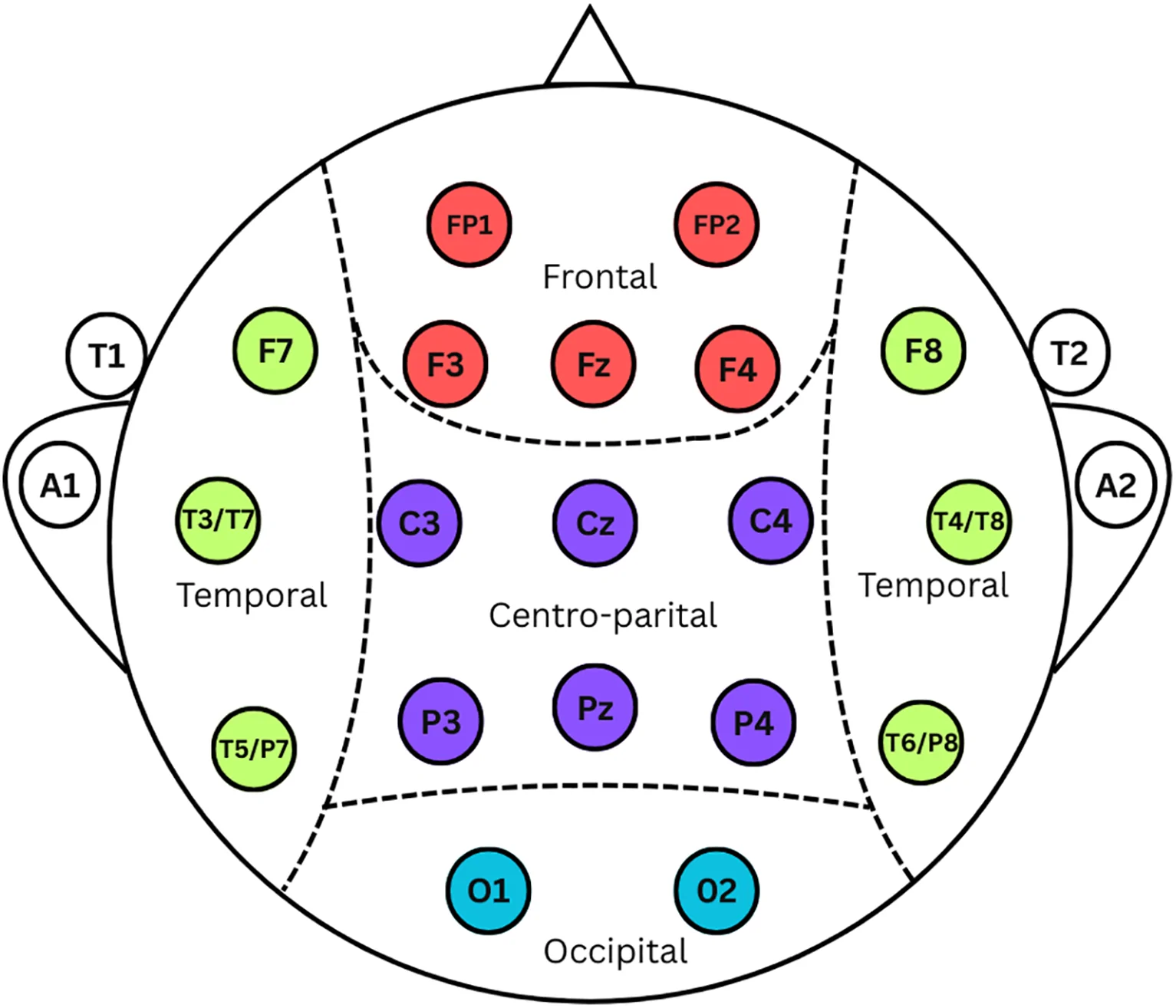
EEG electrode position and the predefined five spatial categories of IED. The “Fp” refers to frontopolar, “F” to frontal, “C” to central, “P” to parietal, “O” to occipital, and “T” to temporal locations.

**Fig 3:**
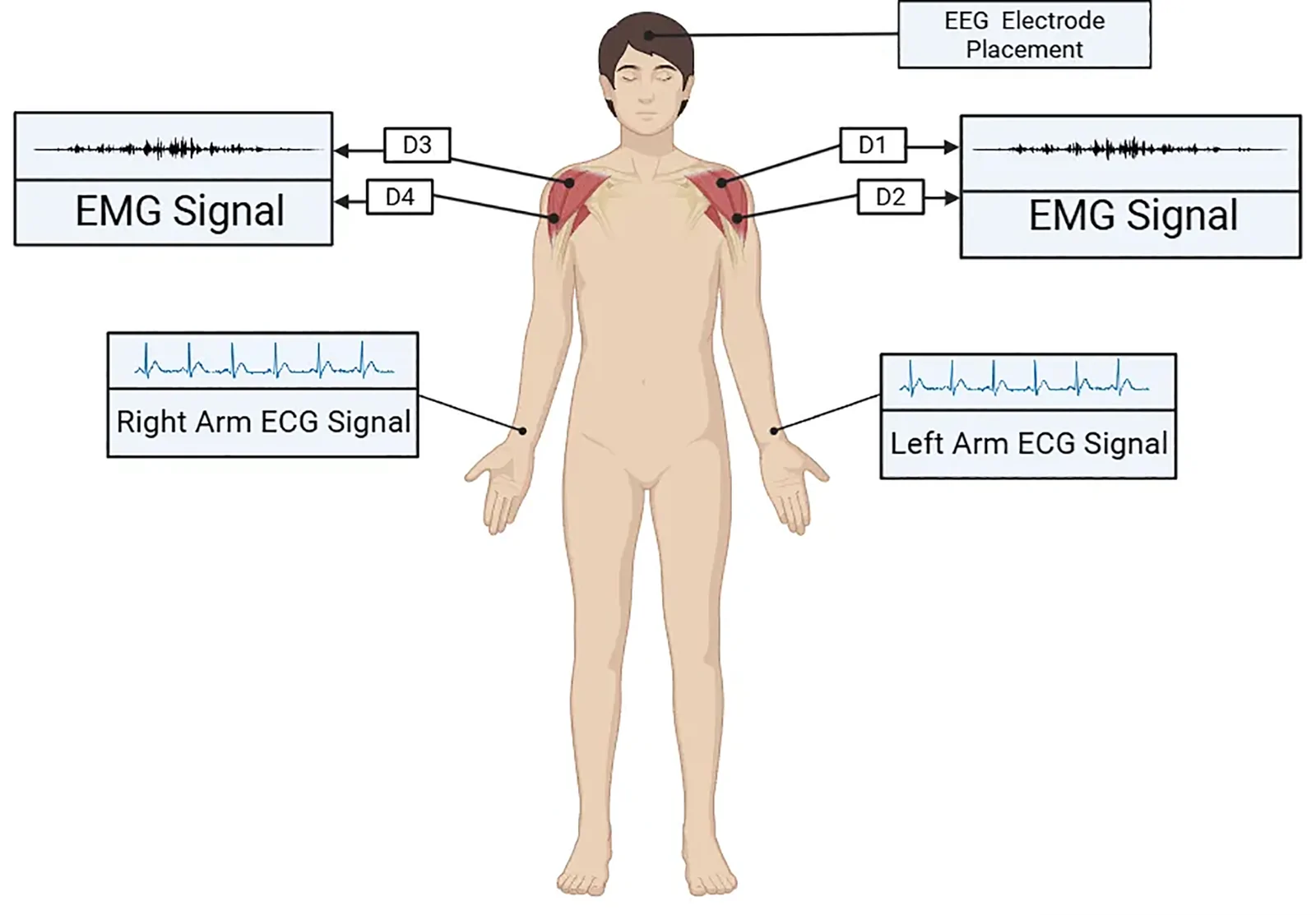
For an electrode arrangement in the ECG and EMG. The right and left arm ECGs are called RA and LA. Bilateral Deltoid EMG channels D1-D4 are indicated.

There are 29 channels on the recordings: 19 scalp EEG channels positioned using the international 10–20 system illustrated in Fig 2, four scalp referential channels (T1, T2, A1, and A2), 2 channels for ECG (RA and LA), and 4 channels for deltoid EMG (bilateral) depicted in Fig 3. Table 1 lists the channel groups and the location where they were recorded. The ECG RA and ECG LA are referred to as lead24 and lead25 in the EEG with ECG SHAP outputs, while this was the original feature naming convention.

**Table 1:** Recording of various signal types including the position of electrodes.

Type of recording electrodes	Number of Electrodes	Position of the Channel
EEG	19	Fp1, F3, C3, P3, O1, F7, T3, T5, Fz, Cz, Pz, Fp2, F4, C4, P4, O2, F8, T4, T6
Referential	4	T1, T2, A1, A2
ECG	2	Right Arm (RA) and Left Arm (LA)
EMG	4	Deltoid muscles: Two channels from the right deltoid region and two channels from the left deltoid region (bilateral).

The open dataset was obtained from the Epilepsy Center of Peking Union Medical College Hospital [5] and contains recordings from 84 participants. Fifty-two recordings contained at least one expert-confirmed IED, whereas 32 recordings were reported as normal EEG. Each participant contributed 20 minutes of recording, giving 28 hours in total. The source dataset contains 22,933 four-second epochs not labelled as IEDs and 2,516 four-second epochs labelled as IEDs. During quality control, two IED epochs were discarded, leaving 2,514 expert-confirmed IED epochs for the present analysis. Non-IED epochs were not included as a target class because the study was designed specifically to evaluate conditional categorization after IED presence had already been established.

The recordings and epoch labels were provided in MATLAB and NumPy formats. Table 2 summarizes the consciousness state of the original 2,516 epochs labelled as IEDs.

**Table 2:** Consciousness state across IED epochs at 4 sec.

Consciousness state	IED epochs	Percentage
Wake	764	30.36%
Sleep	1752	69.63%
Total	2516	100%

### EEG signal preprocessing

The four-second epochs were linearly detrended, notch filtered at 50 Hz and 100 Hz, and band-pass filtered between 0.5 and 40 Hz using a Butterworth filter [15]. Each channel configuration underwent the same applicable preprocessing before feature-matrix construction.

The composite criterion of robust log-variance outliers and low interchannel correlation was used to identify noisy or defective EEG channels. Detected bad EEG channels were interpolated from neighboring channels [13]. Independent component analysis was only used on the EEG channels to remove ocular artifacts while retaining the ECG and EMG signals [14,16]. All scalp EEG channels were then re-referenced to a common average.

Two epochs of wake state were rejected due to the peak-to-peak amplitude being above a strong median absolute deviation (MAD) threshold. The final dataset therefore contained 2,514 IED epochs: 762 wake epochs (30.31%) and 1,752 sleep epochs (69.69%).

### Feature extraction

For each retained four-second epoch, the same database of 26 handcrafted descriptors was computed independently for every included channel, yielding 494 variables for EEG only, 546 for EEG with ECG, and 754 for the full 29-channel input (scalp EEG, referential, ECG, and EMG). The features were grouped into time-domain, frequency-domain, and nonlinear categories. The same descriptor set was intentionally used across EEG and ECG channels to keep the representation fixed during channel-set ablation: if modality-specific feature engineering were introduced only for ECG, any performance change would reflect both the added channels and a changed feature representation. This design choice does not imply that EEG and ECG have equivalent physiology or that every descriptor has the same physiological interpretation across modalities; it provides a controlled, modality-agnostic statistical representation for the comparative analysis.

#### Time-domain features.

The variables used in time domain are mean, median, standard deviation, skewness, kurtosis, peak-to-peak amplitude, waveform length, slope sign changes, Hjorth mobility and Hjorth complexity.

#### Frequency-domain features.

Welch power spectral density estimates were used to compute frequency domain variables. Absolute band power was calculated for each band: delta (0.5–4 Hz), theta (4–8 Hz), alpha (8–13 Hz) and beta (13–30 Hz) as well as the ratio of each band power, peak frequency and median frequency.

#### Nonlinear features.

Nonlinear variables were approximate entropy, sample entropy, permutation entropy, spectral entropy, correlation dimension, detrended fluctuation analysis and the Hurst exponent [17].

### Data splitting

The 2,514 IED-labelled epochs were partitioned using a stratified 80:10:10 epoch-level split which contains 2,011 training epochs, 251 validation epochs, and 252 held-out test epochs. Class proportions were maintained, and identical epoch assignments were used across all channel configurations and classifiers. The partition was not patient-disjoint, so epochs from the same participant could occur in more than one subset. Consequently, the evaluation measures internal held-out-epoch performance and cannot establish generalization to unseen patients. A patient-disjoint or leave-one-patient-out design would be required for that stronger claim. Table 3 summarizes the distribution of the five IED categories in the retained dataset.

**Table 3:** Distribution of IEDs across five anatomical regions in the dataset.

IED Region	Count	Percent (%)
Temporal	700	27.844%
Generalized	573	22.792%
Frontal	458	18.218%
Occipital	417	16.587%
Centro-parietal	366	14.558%
Total	2514	100.000%

### Addressing class imbalance with SMOTE analysis

Synthetic Minority Over-sampling Technique (SMOTE) was applied only to the training subset to reduce imbalance among the five IED categories [18]. Validation and test epochs were not resampled. Applying SMOTE after the split prevented synthetic information from entering the validation or test sets.

For a minority-class feature vector $x_{i}$, a synthetic vector was generated by interpolation between $x_{i}$ and a randomly selected nearest neighbour x_nn from the same class:


$$\begin{array}{c} x_{new}=\;x_{i}+\;\lambda \cdot (x_{nn}-\;x_{i}) \end{array}$$ (1)

where:

- $x_{i}$ is the original minority-class sample;
- $x_{nn\;}$ is a randomly selected nearest neighbour from the same class;
- Lambda λ is sampled from a uniform distribution on [0,1]; and
- $x_{new}\;$ is the synthetic sample on the line segment joining $x_{i}\;$and $x_{nn}$.

### Machine learning classifier evaluation

Machine learning was used here as a comparative framework for mapping the high-dimensional handcrafted feature vectors to five conditional spatial labels. The evaluated classifiers were XGBoost, LightGBM, CatBoost, multinomial logistic regression, linear discriminant analysis (LDA), decision tree, extra trees, random forest, and support vector machine (SVM). Including linear, tree-based, ensemble, and boosting approaches allowed the analysis to assess whether channel-set patterns were specific to one model family. Random-forest results were available for the EEG-only and EEG with ECG analyses, while eight classifiers had results for all three primary channel configurations. The same procedure and partitions were used for each ablation. Hyperparameters were selected using the validation set, and final performance was reported on the held-out test set.

The performance was summarized in terms of overall accuracy, class-specific precision, recall and F1 score along with macro-averaged F1 score [16]. Macro-F1 was highlighted due to the fact that it gives equal importance to each spatial category irrespective of the class frequencies.

### Channel-set ablation design

The channel-set ablation compared feature matrices derived from identical IED epochs and an unchanged feature-extraction pipeline. The EEG-only matrix contained 26 features from each of 19 scalp EEG channels. The EEG with ECG matrix added both ECG RA and ECG LA channels. The full matrix contained all 29 recorded channels: 19 scalp EEG, four referential, two ECG, and four EMG channels. Labels, split assignments, training-only SMOTE, classifier families, and evaluation procedures were held constant. Thus, the EEG-versus-EEG with ECG comparison describes the performance change associated with making ECG-derived variables available to the model. The EEG with ECG-versus-full comparison reflects the joint addition of four referential and four EMG channels and cannot isolate either group independently.

Performance differences between channel configurations are reported descriptively. Formal paired hypothesis tests and confidence intervals were not calculated because the epoch-level prediction outputs needed for paired error analysis were not retained for every configuration. The reported percentage-point differences therefore must not be interpreted as statistically significant effects, and the study does not claim that ECG provides a statistically validated improvement. Inferential testing using retained paired predictions or repeated patient-disjoint resampling is required to establish reproducible performance differences [19].

An additional exploratory EEG with EMG configuration, comprising the 19 scalp EEG channels and four bilateral deltoid EMG channels, was evaluated as a supplementary analysis. The same preprocessing, feature-extraction, data-partitioning, SMOTE based training, classifier-evaluation, and SHAP-analysis procedures were applied. Detailed results are provided in S1 File.

### SHapley Additive exPlanations (SHAP) explainability analysis

SHAP values were used to describe how strongly each channel-feature variable contributed to the outputs of a fitted model [9]. Global importance was calculated as the mean absolute SHAP value across test epochs and output classes and was then normalized to percentages within each feature set. This analysis complements, rather than replaces, the channel ablation: ablation addresses whether performance changes when channels are added, while SHAP identifies where the fitted model places predictive dependence after those channels are available. The normalized percentages are neither statistical effect sizes nor physiological effect sizes. They should not be compared between configurations as though they reflected the same causal quantity since they are relative model-attribution measures inside a certain dataset, feature space, classifier, and target.

#### Shapley value for a feature *i.*


$$\begin{array}{c} \varphi_{i}=\frac{1}{\left|s\right|}\sum_{s⊑Nitbf\{i\}}(f\left(S\cup \left\{i\right\}\right)-f\left(S\right)) \end{array}$$ (2)

- For feature i, the Shapley value is the weighted average change in model output produced by adding that feature to all possible subsets of the remaining features.
- The calculation considers feature subsets that do not contain i and compares the prediction before and after i is added.
- The difference between these predictions is the marginal contribution of feature i.
- Weighted averaging across subsets yields the Shapley value.

#### Multi-Class SHAP (per sample and class).


$$\begin{array}{c} \phi_{i,c\;}=f_{c}\left(S\cup \left\{i\right\}\right)-f_{c\;}(S) \end{array}$$ (3)

For multi-class classification, SHAP values are obtained for each sample, feature, and output class. A positive or negative SHAP value indicates whether the feature shifts the model output toward or away from a class. Class-specific SHAP values were converted to absolute magnitudes for global importance summaries. This formulation extends Shapley attribution to the five-class output.

#### SHAP importance for featurei.


$$\begin{array}{c} \text{SHAP Importance for feature }i=\frac{\text{1}}{N\times C}\sum_{n=\text{1}}^{N}\sum_{c=\text{1}}^{C}\left|\phi_{n,c,i}\right| \end{array}$$

The overall significance of feature has been taken as interest here rather than a single sample or class. Thus, the absolute SHAP values are averaged across all N samples (data points) all c classes. Taking the absolute value makes sure both positive influence and negative influence are counted as importance. This gives a global importance score for each feature.

#### Normalization of SHAP Importance.


$$\begin{array}{c} \text{Normalized SHAP Importance for feature }i=\frac{\text{SHAP Importance for feature i}}{\sum_{j}\text{SHAP Importance for feature j}}\times \text{1}\text{0}\text{0} \end{array}$$

- The raw SHAP importance values might be arbitrary numbers.
- To make them comparable, we scale them so that:
- Now each feature’s importance is expressed as a percentage of total importance.

### Model performance metrics

Model performance was derived from the multi-class confusion matrix. For each class, true positives, false positives, and false negatives were defined using a one-versus-rest formulation. Overall accuracy measured the proportion of correctly classified epochs.

- True positives (TP): epochs of a class correctly assigned to that class.
- True negatives (TN): epochs outside a class correctly not assigned to that class.
- False positives (FP): epochs from other classes incorrectly assigned to the class.
- False negatives (FN): epochs of the class incorrectly assigned elsewhere.

Precision, recall, and F1-score were calculated separately for each class.

- Accuracy was calculated as the number of correct predictions divided by the total number of test epochs.

$$\begin{array}{c} \text{Accuracy}=\;\frac{\left\{\text{TP }+\text{ TN}\right\}}{\left\{\text{TP }+\text{ TN }+\text{ FP }+\text{ FN}\right\}} \end{array}$$ (4)

- Precision was calculated as TP/(TP+FP) and measures the proportion of predictions for a class that were correct.

$$\begin{array}{c} \text{Precision }=\frac{\left\{\text{TP}\right\}}{\left\{\text{TP }+\text{ FP}\right\}} \end{array}$$ (5)

- Recall was calculated as TP/(TP+FN) and measures the proportion of epochs from a class that were correctly identified.

$$\begin{array}{c} \text{Recall }=\frac{\left\{TP\right\}}{\left\{TP+FN\right\}} \end{array}$$ (6)

- F1-score was calculated as the harmonic mean of precision and recall. Macro-F1 was the unweighted mean of the five class-specific F1-scores.

$$\begin{array}{c} \text{F1 }=\text{2}\times \frac{Precision\times Recall}{Precision+Recall} \end{array}$$ (7)

Together, these metrics summarize overall and class-balanced conditional classification performance.

### Ethical approval

This secondary analysis used a publicly available de-identified dataset and involved no new participant recruitment or data collection; additional institutional ethics approval was therefore not required.

## Results

Class-wise metrics were calculated for training, validation, and held-out test subsets. Model selection used the validation set, while principal conclusions are based on the untouched test set. Table 4 summarizes the original full 29-channel results. Tables 5 and 6 compare test accuracy and macro-F1 across channel configurations, and Table 7 reports CatBoost class-specific F1-scores. All between-configuration differences are descriptive.

**Table 4:** Full 29-channel test-set precision, recall, F1-score, and overall accuracy.

Metric	IED spatial category	XGB	LGB	CatB	LR	LDA	DT	ET	SVM
Precision	Generalized	0.960	0.941	0.982	0.800	0.959	0.767	0.926	0.708
	Frontal	0.830	0.830	0.894	0.700	0.796	0.592	0.780	0.500
	Temporal	0.880	0.892	0.943	0.870	0.857	0.848	0.873	0.780
	Occipital	0.884	0.884	0.975	0.939	0.975	0.861	0.974	0.778
	Centro-parietal	0.973	0.973	0.923	0.850	0.946	0.780	0.897	0.875
Recall	Generalized	0.828	0.828	0.948	0.828	0.810	0.793	0.862	0.793
	Frontal	0.848	0.848	0.913	0.761	0.848	0.630	0.848	0.565
	Temporal	0.943	0.943	0.943	0.857	0.943	0.800	0.886	0.557
	Occipital	0.905	0.905	0.929	0.738	0.929	0.738	0.881	0.833
	Centro-parietal	1.000	1.000	1.000	0.944	0.972	0.889	0.972	0.972
F1 Score	Generalized	0.889	0.881	0.965	0.814	0.879	0.780	0.893	0.748
	Frontal	0.839	0.839	0.903	0.729	0.821	0.611	0.812	0.531
	Temporal	0.910	0.917	0.943	0.863	0.898	0.824	0.879	0.650
	Occipital	0.894	0.894	0.951	0.827	0.951	0.795	0.925	0.805
	Centro-parietal	0.986	0.986	0.960	0.895	0.959	0.831	0.933	0.921
Overall Accuracy		0.9008	0.9008	0.9444	0.8254	0.8968	0.7698	0.8849	0.7183

**Table 5:** Test accuracy across channel configurations and descriptive differences (pp, percentage points).

Classifier	EEG only (%)	EEG with ECG (%)	Full 29-channel (%)	ECG gain vs EEG (pp)	Full gain vs EEG with ECG (pp)
CatBoost	86.90	93.25	94.44	+6.35	+1.19
LightGBM	85.71	88.10	90.08	+2.39	+1.98
XGBoost	84.92	88.89	90.08	+3.97	+1.19
Logistic Regression	76.98	79.37	82.54	+2.39	+3.17
LDA	88.89	90.87	89.68	+1.98	−1.19
Decision Tree	77.38	79.76	76.98	+2.38	−2.78
Extra Trees	82.54	86.51	88.49	+3.97	+1.98
SVM	57.94	63.49	71.83	+5.55	+8.34

**Table 6:** Test macro-F1 across channel configurations.

Classifier	EEG only	EEG with ECG	Full 29-channel
CatBoost	0.872	0.933	0.944
LightGBM	0.861	0.883	0.903
XGBoost	0.853	0.890	0.904
Logistic Regression	0.769	0.794	0.826
LDA	0.896	0.913	0.902
Decision Tree	0.775	0.797	0.768
Extra Trees	0.835	0.871	0.888
SVM	0.591	0.646	0.731

**Table 7:** CatBoost class-specific test F1-scores across channel configurations.

IED spatial category	EEG only	EEG with ECG	Full 29-channel
Generalized	0.865	0.939	0.965
Frontal	0.792	0.903	0.903
Temporal	0.875	0.935	0.943
Occipital	0.911	0.916	0.951
Centro-parietal	0.919	0.973	0.960

For the full 29-channel configuration, CatBoost produced the highest test accuracy (94.44%) and macro-F1 (94.44%). Its class-specific F1-scores were 0.965 for generalized, 0.903 for frontal, 0.943 for temporal, 0.951 for occipital, and 0.960 for centro-parietal epochs. These values quantify conditional categorization among expert-confirmed IED epochs and are not measures of IED detection.

### Channel-set ablation results

Adding ECG produced numerically higher held-out test accuracy for all eight classifiers evaluated across the three primary configurations. CatBoost increased from 86.90% with EEG only to 93.25% with EEG with ECG, a descriptive difference of 6.35 percentage points. The full 29-channel CatBoost result was 94.44%, a further descriptive increase of 1.19 percentage points. The full configuration exceeded EEG with ECG for six classifiers, whereas LDA and decision tree performed better with EEG with ECG. Thus, the fixed-split results show a consistent numerical association between ECG inclusion and higher internal epoch-level accuracy, while the effect of the remaining channel groups was model-dependent. Because paired inferential testing was not performed, these differences are not presented as statistically significant improvements.

Detailed validation- and test-set results for the EEG with EMG, EEG-only, and EEG with ECG configurations are provided in S1 File. These supplementary results include class-specific precision, recall, and F1-score, together with overall classification accuracy for the evaluated classifiers. Table 5 depicts the test accuracy across channel configurations and descriptive differences for EEG signals only, EEG with ECG signals and full 29 channel signals while Table 6 summarizes macro-F1 across channel configurations of test set. And finally, Table 7 summarizes CatBoost class-specific test F1-scores across different channel configurations

Relative to EEG-only CatBoost, EEG with ECG yielded higher F1-scores for generalized, frontal, temporal, occipital, and centro-parietal categories. The full configuration yielded the highest generalized, temporal, and occipital F1-scores; EEG with ECG yielded the highest centro-parietal F1-score; and frontal F1 was 0.903 in both ECG-containing configurations. These observations describe the fixed test split and were not subjected to formal inferential testing.

### EEG with ECG SHAP analysis

Within the EEG with ECG CatBoost model as stated in Table 8, ECG RA and ECG LA received 15.79% and 15.12% of normalized global SHAP attribution, respectively. Together they accounted for 30.90% of normalized attribution in that feature set. Beta-band power was the leading feature family (18.76%), followed by peak-to-peak amplitude (9.72%), theta power (8.08%), alpha-beta ratio (7.98%), and alpha power (7.28%). Table 8 lists the largest individual channel-feature attributions.

**Table 8:** Largest CatBoost EEG with ECG channel-feature SHAP attributions on the test set.

Rank	Channel	Feature	SHAP importance (%)
1	ECG RA (lead24)	Alpha-beta ratio	3.787
2	Fp1 (lead1)	Beta power	3.329
3	ECG LA (lead25)	Alpha power	2.397
4	ECG LA (lead25)	Theta-alpha ratio	2.314
5	Fz (lead9)	Peak-to-peak amplitude	2.066
6	Fz (lead9)	Theta power	2.047
7	ECG LA (lead25)	Beta power	2.029
8	ECG LA (lead25)	Alpha-beta ratio	1.856
9	T5 (lead8)	Beta power	1.848
10	F3 (lead2)	Beta power	1.527

Within the EEG with ECG using CatBoost model, the two ECG channels received substantial normalized attribution, indicating that the fitted classifier used ECG-derived variables when separating the five expert-assigned labels. This attribution pattern does not demonstrate a clinically meaningful interictal biomarker or an autonomic localization signal. Patient identity, cardiac morphology, sleep-wake state, recording conditions, movement, contamination, and other dataset-specific structure remain plausible alternative explanations, which is precisely why the SHAP analysis is interpreted as a model-dependence analysis rather than physiological validation.

### SHAP feature-importance analysis

Table 9 summarizes aggregated SHAP attribution for the full 29-channel models. The values identify variables used by the trained classifiers in the conditional five-class task; they do not show that a feature independently improved accuracy or represents a validated biomarker.

**Table 9:** Aggregated feature SHAP importance across classifiers for the full 29-channel configuration.

Features	CatBoost	LGBM	XGB	DT	ET	RF	LR	LDA
Power Beta	15.64	22.27	24.03	24.03	5.53	11.10	2.27	2.15
Peak-to-Peak	10.60	11.12	11.86	11.86	10.34	11.35	1.07	4.09
Power Alpha	4.97	7.39	6.62	6.62	4.69	6.75	4.78	2.32
Hjorth Mobility	2.85	4.16	4.03	4.03	6.18	5.72	1.96	3.76
Power Theta	6.20	8.90	9.27	9.27	6.15	9.87	4.23	5.88
Waveform Length	7.18	1.66	2.40	2.40	8.17	7.14	1.63	6.02
Approximate Entropy	4.81	5.04	4.27	4.27	8.48	6.87	4.31	3.13
Standard Deviation	5.52	1.86	2.66	2.66	9.80	9.33	1.91	10.80
Alpha-Beta Ratio	6.45	4.51	5.27	5.27	3.81	3.61	7.33	1.34
Skewness	4.21	4.27	3.48	3.48	1.65	1.96	2.81	0.57
Theta-Alpha Ratio	5.19	3.12	2.92	2.92	2.77	2.67	1.78	3.91
Permutation Entropy	2.05	3.82	4.38	4.38	4.24	2.61	2.33	6.55
Kurtosis	3.94	3.50	3.15	0.77	1.97	1.66	3.98	1.00
Correlation Dimension	3.66	5.35	4.66	4.66	2.39	1.31	9.52	0.56
Sample Entropy	2.19	2.47	1.67	1.67	3.75	2.73	3.72	1.19
Power Delta	1.50	0.07	0.25	0.25	3.84	4.77	1.08	3.23
Hjorth Complexity	1.70	1.34	0.77	0.77	1.74	1.51	3.23	2.09
Sample Shannon’s Entropy	2.19	2.08	2.13	2.13	4.29	2.33	4.67	6.59
Median Frequency	1.12	0.87	0.75	0.75	1.48	0.87	3.23	0.88
Detrended Fluctuation Analysis	1.83	0.47	0.57	0.57	3.46	2.06	1.43	2.57
Hurst Exponent	1.08	0.61	0.39	0.39	0.73	0.47	1.78	0.83
Spectral Entropy	1.21	1.37	0.79	0.79	2.19	1.09	1.72	1.28
Delta-Theta Ratio	1.60	1.34	1.68	1.68	1.02	1.03	6.30	1.07
Peak Frequency	1.02	1.41	1.34	1.34	0.66	0.52	1.26	0.34
Median	1.41	0.79	0.59	0.59	0.54	0.50	4.33	0.42
Mean	0.25	0.20	0.10	0.10	0.12	0.16	4.07	27.43

Table 10 reports channel-wise SHAP attribution for the full 29-channel configuration.

**Table 10:** Channel-wise SHAP importance across classifiers for the full 29-channel configuration.

Lead	Indication	CatBoost	LGBM	XGB	DT	ET	RF	LR	LDA
Lead 1	EEG Fp1	5.44	7.48	7.91	7.91	5.10	5.63	4.48	4.09
Lead 2	EEG F3	5.96	4.53	3.64	3.64	3.67	4.29	4.07	3.39
Lead 3	EEG C3	3.30	2.41	1.45	1.45	4.50	5.29	3.58	3.23
Lead 4	EEG P3	5.82	6.38	6.41	6.41	5.83	6.60	6.28	3.50
Lead 5	EEG O1	1.45	0.02	0.10	0.10	1.94	0.68	1.92	3.04
Lead 6	EEG F7	0.59	1.12	1.55	1.55	3.25	2.90	1.46	2.82
Lead 7	EEG T3	1.19	1.89	2.50	2.50	3.80	2.45	2.91	2.72
Lead 8	EEG T5	1.92	2.74	3.18	3.18	3.32	2.39	4.27	4.08
Lead 9	EEG Fz	6.68	6.47	6.68	6.68	6.69	9.73	3.39	3.90
Lead 10	EEG Cz	3.30	2.93	2.18	2.18	4.23	4.89	4.17	3.85
Lead 11	EEG Pz	1.53	0.84	0.62	0.62	1.48	1.12	2.77	3.00
Lead 12	EEG Fp2	1.55	0.57	0.89	0.89	2.04	1.26	2.28	3.28
Lead 13	EEG F4	0.46	0.08	0.07	0.07	1.46	0.84	3.93	2.63
Lead 14	EEG C4	1.62	1.25	1.42	1.42	2.08	1.97	1.19	2.61
Lead 15	EEG P4	3.59	2.10	2.18	1.77	4.74	4.49	2.59	3.43
Lead 16	EEG O2	1.67	0.84	0.76	0.76	2.11	1.07	2.29	4.08
Lead 17	EEG F8	6.80	5.96	6.77	6.77	7.09	8.84	3.73	3.73
Lead 18	EEG T4	1.97	1.03	1.06	1.06	3.29	2.16	4.55	2.58
Lead 19	EEG T6	3.64	2.42	2.62	2.62	3.44	3.05	2.20	2.94
Lead 20	EEG T1	3.15	4.83	4.48	4.48	3.85	3.12	5.30	2.95
Lead 21	EEG T2	1.65	0.96	1.14	1.14	3.04	1.92	4.44	2.34
Lead 22	EEG A1	1.36	1.40	0.97	0.97	1.74	1.14	2.60	1.95
Lead 23	EEG A2	1.66	0.69	0.63	0.63	2.55	2.13	2.90	2.04
Lead 24	ECG RA	11.18	13.14	12.53	12.53	5.29	5.33	3.14	4.03
Lead 25	ECG LA	10.53	18.41	18.86	18.86	5.36	8.09	5.82	2.60
Lead 26	EMG (Left deltoid 1)	3.92	2.90	3.18	3.18	2.26	2.69	4.97	4.15
Lead 27	EMG (Left deltoid 2)	3.04	1.07	1.52	1.52	2.03	2.46	4.71	4.03
Lead 28	EMG (Right deltoid 1)	2.16	3.10	2.76	2.76	2.10	1.76	2.09	8.75
Lead 29	EMG (Right deltoid 2)	2.94	2.44	2.34	2.34	1.71	1.70	1.96	4.28

CatBoost achieved the highest full-channel test accuracy. Table 11 lists its ten largest combined channel-feature attributions.

**Table 11:** Top combined channel-feature SHAP importance values for the full-channel CatBoost model.

Rank	Feature	SHAP Importance (%)
1	lead9_waveform_length	2.68
2	lead25_theta_alpha_ratio	2.20
3	lead19_power_beta	2.11
4	lead24_alpha_beta_ratio	2.00
5	lead17_ptp	1.83
6	lead1_power_beta	1.74
7	lead25_power_beta	1.66
8	lead25_power_alpha	1.64
9	lead17_power_beta	1.52
10	lead2_power_beta	1.50

Across the full-channel models in Tables 9–11, spectral-power and waveform-morphology variables frequently received high attribution. Beta power and peak-to-peak amplitude were prominent in several tree-based classifiers. These results indicate that the models used these variables to separate the five expert-assigned labels; they do not establish that the variables detect IEDs or identify their physiological source.

In the full configuration, ECG RA and ECG LA received high attribution in several classifiers, while scalp EEG channels including Fz, F8, and Fp1 also contributed. When combined, the SHAP and ablation analyzes offer two distinct descriptive perspectives: the ablation shows that ECG availability coincided with higher fixed-split accuracy across the eight comparable classifiers, and SHAP shows that the fitted models assigned appreciable predictive attribution to ECG-derived variables. Neither result establishes a causal autonomic mechanism or statistically validated modality benefit, and normalized SHAP percentages can change when the feature set changes.

In EEG-only CatBoost, beta power contributed 17.93% to normalized feature-family attribution and the most dominant channels were Fz (12.18%), F3 (10.74%), Fp1 (9.06%) and F8 (8.34%). In EEG with ECG CatBoost beta power was ranked 18.76%, while ECG RA and ECG LA were the two top ranked channels. These percentages are descriptive features of the fitted models.

Additional SHAP analyses are presented in S1 File, including aggregated feature-family importance, channel-wise importance, and the ten highest-ranked channel-feature attributions for each analysed classifier. These normalized SHAP values represent model-specific dependence within the corresponding feature set and should not be interpreted as physiological effect sizes, validated biomarkers, or evidence of causal mechanisms.

## Discussion

This study addressed a focused downstream question whether after expert confirmation that an epoch contains an IED, can the epoch be classified among five predefined scalp-distribution categories, and does access to routinely co-recorded auxiliary channels alter that classification. Under a common handcrafted-feature and machine-learning pipeline, the five labels were separable on the internal held-out epoch split, and ECG inclusion was associated with numerically higher accuracy for every directly comparable classifier. SHAP then identified the variables on which the fitted models depended, providing an attribution-level complement to the channel-set ablation. These results define the study as a methodological analysis of conditional categorization and multimodal model dependence, rather than an IED detector or clinical localization system.

### Scope and methodological contribution

The principal methodological contribution is the controlled separation of three questions that are often mixed up which includes IED detection, downstream spatial categorization, and interpretation of auxiliary-channel dependence. By fixing IED presence through expert annotation, the analysis isolates discrimination among five scalp-distribution labels. By holding epochs, feature extraction, partitions, training strategy, and classifier procedures constant across channel sets, the ablation asks whether making additional recorded modalities available changes internal classification. This restricted setting is useful for benchmarking downstream annotation models and for revealing whether a classifier may exploit information outside the expected scalp EEG channels. It does not reproduce routine clinical EEG review, in which IED presence is unknown, and it does not constitute source localization.

### Performance of the classifiers in the context of prior work

CatBoost test accuracy was 86.90% with EEG only, 93.25% with EEG with ECG, and 94.44% with the full 29-channel input. The numerical increase associated with adding ECG was larger than the subsequent increase associated with adding the remaining channels for CatBoost, but the pattern was classifier-dependent. LDA and decision tree performed better on EEG with ECG channels than with the full channel set. Because no paired confidence intervals or hypothesis tests were available, these differences are descriptive. The results therefore show a dataset-specific fixed-split association between ECG availability and classification performance and do not establish a statistically significant, universal, causal, or clinically validated benefit of ECG.

Table 12 places the present work alongside representative literature to clarify task and validation differences, not to claim benchmark superiority. Most prior studies address binary IED detection or use different spatial targets, datasets, architectures, and validation strategies; Chung et al. [9], for example, used a patient-disjoint approach for focal IED detection and lobe classification. Because those methods were not reimplemented on the present dataset under the same partitions, their published performance values are contextual only and are not direct baselines for the current conditional five-class task.

**Table 12:** Comparison of the proposed framework with representative studies of IED detection and spatial classification.

Study	Task and channels	Validation	Reported result
Chung et al., 2023 [20]	32-channel EEG; deep-learning focal IED detection and lobe classification	Leave-one-patient-out	Frontal 79.3–86.4%; temporal 93.3–94.2%; occipital 95.5–97.2%
Bagheri et al., 2019 [21]	19-channel EEG; handcrafted-feature binary IED detection	5-fold cross-validation	Sensitivity 96.93%; specificity 78.41%
Thomas et al., 2020 [22]	19-channel EEG; CNN binary IED versus non-IED classification	5-fold cross-validation	Accuracy 81.41%
Tjepkema-Cloostermans et al., 2018 [23]	19-channel EEG; deep binary focal IED detection	Train/test split	AUC 0.94; sensitivity 47.4%; specificity 98.0%
Wang et al., 2023 [24]	19-channel EEG; two-stage binary IED detection	5-fold patient-wise cross-validation plus test set	AUPRC approximately 0.811; F1 approximately 0.745
Fürbass et al., 2020 [25]	19-channel EEG; AI-based binary detection with clustering	Not reported	Sensitivity 89%; specificity 70%; accuracy 80%
Proposed work	19-channel EEG, EEG with ECG, and full 29-channel input; conditional five-class categorization of expert-confirmed IED epochs	Stratified 80:10:10 epoch-level split	Best EEG only: LDA 88.89%; EEG with ECG: CatBoost 93.25%; full: CatBoost 94.44%

Previous studies indicate that performance may decline as epileptiform classification moves from binary decisions to finer spatial categories [6,20]. The present results show that handcrafted-feature classifiers can separate five predefined labels within a curated collection of IED-positive epochs. This observation does not imply that the task is more clinically difficult than binary detection, because non-IED background, artifacts, and IED-like transients were excluded.

Classical and boosting-based methods remained competitive for the structured handcrafted features. CatBoost performed best with the ECG-containing configurations, whereas LDA performed best with EEG only. This configuration-dependent ranking argues against treating any model or channel set as universally superior.

Earlier feature-based studies showed that handcrafted EEG variables can support epileptiform analysis, often in binary detection settings [24]. The contribution of the present analysis is different and narrower: it applies a common handcrafted representation and fixed modelling workflow to quantify how conditional five-class performance changes across scalp EEG, EEG with ECG, and the complete recorded channel set, and then uses SHAP to identify which variables the fitted models depend on. The study does not demonstrate superiority over prior methods because those methods were not benchmarked on this dataset, and it does not test whether ECG improves IED-versus-non-IED detection.

Cross-study comparisons are limited by differences in annotation, class definitions, preprocessing, sampling, validation, channel composition, and target definition. More importantly, the current epoch-level split may place epochs from one participant in multiple subsets, whereas patient-disjoint schemes such as leave-one-patient-out validation are more appropriate for estimating performance on unseen individuals. The reported values should therefore be interpreted only as internal held-out-epoch performance. Direct reimplementation of prior methods on the same dataset and patient-disjoint evaluation would be required for a rigorous comparative or clinical-generalization claim.

### Top features and leads (SHAP analysis)

The channel ablation provides a descriptive performance pattern that complements the SHAP rankings. All eight comparable classifiers achieved numerically higher fixed-split test accuracy with ECG than with scalp EEG alone. This consistency motivates examination of how the fitted models used the added modality, but it is not equivalent to statistical evidence of an ECG effect. ECG was not evaluated for separating IED from non-IED activity, and the full-input comparison cannot identify an independent EMG effect because referential and EMG channels were added together.

SHAP is useful in this design because performance alone cannot show which variables account for a model’s decisions after a channel group is added. By decomposing model outputs into channel-feature attributions, SHAP indicates whether the fitted classifier actually depends on the newly available modality and identifies the features through which that dependence appears. For example, an EEG frontal electrode may rank highly because it helps separate frontal-labelled epochs from other IED-labelled categories, while ECG ratios may contribute to class separation for dataset-specific reasons. These attributions explain the fitted model; they do not validate physiology, detect IEDs, or localize an epileptogenic source.

The prominence of beta power, waveform morphology, and selected EEG and ECG channels is consistent with the models exploiting spectral and amplitude differences among labelled epochs. This compatibility does not prove a physiological explanation. Normalized ECG attribution may also be influenced by feature-space composition. Patient-disjoint validation and controlled confound analyses are required before clinical or biological meaning can be assigned.

### Interpretation of ECG and EMG contributions

High ECG attribution may reflect patient-specific cardiac morphology, sleep-wake state, medication, recording conditions, movement, cross-channel contamination, or other dataset structure; a genuine interictal neurocardiac association is only one possible explanation. The current analysis cannot distinguish among these alternatives. Seizure-related autonomic literature is therefore not treated as direct evidence for ECG-based spatial classification of interictal discharges.

The performance ablation shows that ECG inclusion was associated with improved conditional classification across the tested classifiers, but the mechanism remains unresolved. The full configuration does not isolate EMG because referential and EMG channels were introduced together. ECG SHAP prominence should therefore be regarded as hypothesis-generating rather than proof of an autonomic mechanism, a clinical biomarker, or spatial source information.

### Imbalanced dataset handling

SMOTE was only used on the training subset. Validation and test distributions were left unchanged. This reduced training imbalance but did not eliminate unequal class representation or guarantee equivalent performance across categories.

### Strengths of the study

Strengths include expert-confirmed epoch labels, identical epoch partitions across channel configurations, training-only oversampling, multiple classifier families, class-specific metrics, a staged channel ablation, and model-level interpretation with SHAP. In particular, the design keeps the feature representation and modelling workflow fixed when ECG is added, so the observed descriptive performance differences can be attributed to channel availability within this dataset rather than to a simultaneous change in feature engineering. The paired use of ablation and SHAP also distinguishes the question of whether performance changes from the question of which variables the fitted model uses.

### Limitations

Several limitations define the interpretation of this work. First, only expert-confirmed IED epochs were analyzed; consequently, the study does not evaluate IED detection, specificity, false-positive reduction, artifact rejection, or clinical screening. Second, the epoch-level split was not patient-disjoint. Participant overlap may allow models, particularly ECG-containing models, to exploit patient- or recording-specific information and may yield optimistic estimates relative to unseen-patient evaluation. Third, configuration differences are descriptive because paired prediction outputs were not retained for formal hypothesis tests or confidence intervals; therefore, no statistically significant ECG improvement is claimed. Fourth, prior published methods were not reimplemented on this dataset, so Table 12 provides context rather than a direct performance benchmark and the study does not claim superiority over existing approaches. Fifth, although EEG with ECG isolates the addition of the two ECG channels, the full configuration jointly adds referential and EMG channels, so independent EMG and referential effects remain unknown. Sixth, the same modality independent descriptor library was used for EEG and ECG to preserve a controlled ablation; this supports comparability but does not exploit modality-specific feature engineering. Seventh, spatial information is represented implicitly through channel-specific feature names rather than explicit electrode geometry or topographic maps. Finally, SHAP attributions are model-specific associations and do not establish physiological biomarkers or causal mechanisms.

### Future directions

Future work should prioritize patient-disjoint evaluation, including leave-one-patient-out or comparable subject-level validation, followed by external validation. Retaining subject identifiers and per-epoch prediction outputs would also permit paired confidence intervals, significance testing, and analysis of patient-specific confounding. A rigorous benchmark should reimplement representative prior methods, including approaches mentioned in Table 12, on the same dataset and compare them under identical patient-disjoint partitions, with and without ECG where technically applicable. Additional extensions should include representative non-IED background, artifacts, and IED-like transients; separate ablations of referential, ECG, and EMG channels; modality-specific feature representations tested against the current common-feature baseline; and explicit topographic or graph-based spatial representations. These studies are needed before the descriptive ECG association observed here can be interpreted as reproducible or clinically useful.

## Conclusion

This study demonstrates that conditional five-class categorization of expert-confirmed IED epochs is a separable downstream machine-learning problem within the evaluated dataset and fixed epoch-level split. A controlled channel ablation showed numerically higher test accuracy after ECG was added to scalp EEG for all eight directly comparable classifiers; CatBoost increased from 86.90% with EEG only to 93.25% with EEG with ECG and 94.44% with the full 29-channel input. SHAP analysis further showed that the fitted ECG-containing models placed substantial attribution on ECG-derived variables, helping explain how the models used the added modality. These findings establish a methodological basis for studying auxiliary-channel dependence in conditional IED categorization, but the performance differences are descriptive rather than statistically significant, and no direct benchmark against prior methods was performed. Patient-disjoint validation, paired inferential testing, direct benchmarking, and evaluation that includes non-IED epochs are required before unseen-patient or clinical utility can be assessed.

## Supporting information

S1 File The file contains validation- and test-set analyses for the EEG with EMG, EEG-only, and EEG with ECG configurations, including class-specific performance metrics, aggregated feature-family importance, channel-wise importance, and classifier-specific channel-feature rankings. (DOCX)
